# Validity and reliability of questionnaires measuring physical activity self-efficacy, enjoyment, social support among Hong Kong Chinese children

**DOI:** 10.1016/j.pmedr.2014.09.005

**Published:** 2014-10-16

**Authors:** Yan Liang, Patrick W.C. Lau, Wendy Y.J. Huang, Ralph Maddison, Tom Baranowski

**Affiliations:** a Department of Physical Education, Hong Kong Baptist University, Hong Kong; b National Institute for Health Innovation, University of Auckland, New Zealand; c USDA/ARS Children's Nutrition Research Center, Department of Pediatrics, Baylor College of Medicine, Houston, TX, United States

**Keywords:** Validity, Reliability, Self-efficacy, Enjoyment, Social support, Children

## Abstract

**Background:**

Physical activity (PA) correlates have not been extensively studied in Hong Kong children.

**Objective:**

The aim of this study is to assess the validity and reliability of translated scales to measure PA related self-efficacy, enjoyment and social support in Hong Kong Chinese children.

**Methods:**

Sample 1 (*n* = 273, aged 8–12 years) was recruited (May–June, 2013) from two primary schools. Confirmatory factor analyses (CFA) were conducted to assess factorial validity. Criterion validity was assessed by correlating measured constructs with self-reported PA. Cronbach's alpha was computed to assess scale internal consistency. The intraclass correlation coefficient (ICC) was performed to assess scale test–retest reliability. Criterion validity was further examined in Sample 2 (*n* = 84, aged 8–12 years) from a third school by correlating measured constructs with objectively measured PA collected in September 2013 and February 2014.

**Results:**

The CFA results supported the one-factor structure of the scales. All PA correlates were significantly (*p* < 0.01) associated with self-reported PA in Sample 1. Self-efficacy and enjoyment were significantly (*p* < 0.05) correlated with objectively measured PA in Sample 2. All the scales demonstrated acceptable internal consistency. All ICC values of the scales suggested acceptable test–retest reliability.

**Conclusion:**

The results provide psychometric support for using the scales to measure PA correlates among Hong Kong Chinese children.

## Highlights

• Validity and reliability of translated scales measuring PA correlates were assessed among Hong Kong children.
• Confirmatory factory analyses supported the scale factorial validity.
• Scale criterion validity was supported by significant correlation between each construct with self-reported PA.
• Scale internal consistency and test-rest reliability were all acceptable.
• Scale criterion validity (self-efficacy and enjoyment) was further demonstrated with objectively measured physical activity in a smaller sample.

## Introduction

The benefits of physical activity (PA) for children have been well documented (Biddle et al., 2004, World Health Organization, 2010). However, a large proportion of children do not perform PA at levels necessary to enhance health (Biddle et al., 2004, Troiano et al., 2008), including Chinese children in Mainland China (Wang et al., 2013) and Hong Kong (Sports Commission of Hong Kong Special Administrative Region Government, 2012). Only 9% of Hong Kong primary school children (7–12 years old) accumulated at least 60 min of moderate-to-vigorous physical activity (MVPA) every day (Sports Commission of Hong Kong Special Administrative Region Government, 2012). Thus effective strategies are needed to promote PA among Hong Kong children. Identifying and understanding the factors associated with PA are needed to modify the behavior (Sallis and Owen, 1999).

In western countries psychosocial variables, such as self-efficacy and social support, have been positively associated with children's PA (Van der Horst et al., 2007). Enjoyment of PA was a mediator of the effect of a school-based PA intervention (Dishman et al., 2005). Few measures have been validated of such PA correlates in Chinese. One questionnaire, developed for Taiwanese adolescents, measured PA correlates based on the health promotion model (Wu et al., 2002), and one questionnaire, developed for Hong Kong children, measured psychosocial and environmental correlates (Huang et al., 2011). The latter questionnaire included items to measure self-efficacy, peer support, and family support, but focused on the influence of environmental factors (Huang et al., 2011). The self-efficacy measure focused on children's confidence in finding and creating an environment to support their PA, not a suitable measure for the overall self-efficacy including barriers-efficacy, seeking-support-efficacy, being active despite competitive activities, and engaging in the task of being regularly active. Peer support was only captured by two items (being physically active with me; and offer encouragement to be physically active); and enjoyment was not examined, thereby limiting their use. Studies of PA correlates among children speaking Cantonese Chinese, like the children in Hong Kong, would benefit from having documented validated full scales of self-efficacy, peer and family support, and enjoyment of PA.

This study examined the validity and reliability of instruments to assess PA self-efficacy, PA enjoyment, and social support for PA among Hong Kong Chinese children.

## Methods

### Participants

A sample (Sample 1) of 273 Chinese children (134 girls and 139 boys) in grades 3–6 were recruited from two Hong Kong Government primary schools. An additional sample (Sample 2) of 84 children (33 girls and 51 boys) in grades 4–6 was recruited from a third similar school to further examine the criterion validity of the scales. All participants had no restrictions, such as physical or psychological limitations, to engage in PA. Both participants and their guardians provided written informed consent. The study was approved by the Senate Committee on the Use of Human and Animal Subjects in Teaching and Research, Hong Kong Baptist University.

### Tested questionnaires

An 8-item scale was used to measure PA self-efficacy (see Appendix). The items were taken from a previously validated instrument (Motl et al., 2000), and modified based on a published simpler version (Ward et al., 2007, p.243). The modifications included: 1) shortening the items, 2) specifying video game as sedentary video game, since active video game could lead to light to moderate PA (Liang and Lau, 2014) and has been positively associated with self-reported PA (Simons et al., 2012), and 3) replacing one item (“I have the coordination I need to be physically active during my free time on most days”) with another one (“I can do active things because I know how to do them”). These modifications were based on the target population being younger than the previous study (Motl et al., 2000). Each item used a Likert scale ranging from 1 (“Disagree a lot”) to 5 (“Agree a lot”).

A 5-point Likert scale (Ward et al., 2007, p.244) was used to measure PA enjoyment ranging from 1 (“Disagree a lot”) to 5 (“Agree a lot”). All 7 items (e.g., “When I am active, I feel bored”) were negatively worded; thus higher scores indicated lower PA enjoyment. These 7 items were taken from a modified 16-item version (Motl et al., 2001) of Physical Activity Enjoyment Scale (PACES) (Kendzierski and DeCarlo, 1991). The other 9 items were all positively worded, and were found associated with an irrelevant methodologic effect which may explain the lack of support for the unidimensional structure of the original PACES (Motl et al., 2001).

Ten items adapted from the Social Support for Exercise Scale (Sallis et al., 1987) were used to measure social support for PA. The word “exercise” was changed to “PA” in the present study. Although the scale was developed for college students, the 10 items have been used in a younger population (12.55 ± 0.65 years old) (Hsu et al., 2011). Children rated how often they received PA related social support (e.g., “During the past three months, my family or friends discussed PA with me”) on a 5-point scale (1 = none to 5 = very often). There was also a rating of “do not apply”.

No culture-specific items were identified (judged by LY and LPWC) in the English items. Therefore all the items were translated from English to Chinese by two bilingual Chinese researchers (two Ph. D. students who have experience in PA epidemiology and translating surveys from English to Chinese), and consensus was achieved by discussion with a bilingual expert panel (including the translators, two researchers in exercise science, and two authors LY and LPWC). LPWC made all final decisions based on the discussion. Back-translation was then conducted by an independent translator. A satisfactory version was reached by discussion of the panel. Cognitive interviews were conducted with a separate sample (*n* = 16) of similar age from one of the participating schools. Participants were asked whether they completely understood the items in the translated questionnaires, and if not, which of the alternative expressions sounded more understandable. Minor changes (e.g., because children did not understand “does not apply” in the social support scale, so this rating was excluded) were made by the expert panel based on the responses from these interviews. This multistep approach was conducted for the translation because it “could provide a series of filters that function in tandem to ferret out both obvious and subtle defects in the questions” (Willis et al., 2010). The specific items of each scale are provided in the appendix.

### Children's physical activity behaviors

To test scale criterion validity, a previously validated questionnaire (Children's Leisure Activities Study Survey questionnaire-Chinese version, CLASS-C) (Huang et al., 2009), was completed by participants. Children reported the total time they spent in PA in the past week with a checklist of activities during weekdays and weekend days. The questionnaire was scored for the daily time spent in MVPA (Huang et al., 2011).

Objectively measured PA was assessed in Sample 2 using the triaxial GT3X accelerometer (ActiGraph Ltd, Pensacola, FL, USA). Children were instructed to wear the accelerometer on their right hip during waking hours, except during bathing, water sports or contact sports for 7 consecutive days. If the wearing time was 480 min or more a day, that day was completed. Non-wear time was defined as 20 min or more of consecutive 0 s. To be included in the analyses, subjects needed to provide at least 2 completed weekdays and 1 completed weekend day. Age-specific cut-off points were used to determine the minutes of MVPA (Freedson et al., 2005). The average minutes of daily MVPA was computed. In addition, vector magnitude (VM) counts per minute (cpm) was also derived to reflect the overall PA (Yildirim et al., 2011).

### Height and weight

In Sample 1, children reported their heights and weights in questionnaires. In Sample 2, standing heights were measured to the nearest 0.1 cm using a portable stadiometer (Seca, Model 214, Hamburg, Germany). Weight (measured to the nearest 0.1 kg) was assessed using an electronic scale (Tanita, Model TBF-410GS, Tokyo, Japan). Body mass index (BMI) was computed (weight in kilograms divided by height in meters squared), and BMI percentiles were computed based on an international sample as recommended by Cole and Lobstein (2012).

### Procedures

The main study was conducted from May to June, 2013. Children (Sample 1) were surveyed during physical education classes or school recess, and were encouraged to ask questions as necessary. To assess test–retest reliability, the questionnaires were administered on two occasions 7 days apart. Each time took approximately 15–20 min for participants to complete the questionnaires. The criterion validity of the scales was further examined in Sample 2 by correlating the correlates with the objectively measured PA collected in September 2013 (*n* = 49) and February 2014 (*n* = 35). The two rounds of data collection is because the objectively measured PA data were collected for another school-based PA intervention study, for which baseline data were collected at the beginning of two semesters as specified above. Children who would like to take part in the intervention study and provided both self-reported PA and objectively measured PA data at baseline, no matter whether they met the inclusion criteria for the intervention study, were included in Sample 2.

### Data analysis

Scale factorial validity was tested by confirmatory factor analyses (CFA) using Amos 21.0 (IBM Inc. Armonk, NY) with the maximum likelihood model. The sample size (Sample 1) was adequate to conduct CFA since the ratio of the sample size to the number of freely estimated parameters was greater than 10:1 (Bentler and Chou, 1987). Since the four scales were supposed to be unidimensional, the one-factor model was assessed for each scale using data from the first administration. Model fit was assessed using the chi square statistic, the Comparative Fit Index (CFI, > 0.9) (Bentler, 1992), the root mean square error of approximation (RMSEA, < 0.08) (Browne and Cudeck, 1993), and the standardized root mean square residual (SRMR, ≤ 0.08) (Schreiber et al., 2006).

Cronbach's alpha was computed for each scale to assess internal consistency with 0.7 considered minimally acceptable (Nunnaly, 1978). Ratings of each scale were averaged, and scale test–retest reliability was assessed by an intraclass correlation coefficient (ICC). If one value was missing for a subject, the average score was computed on the remaining items. If more than one value was missing, the participant was excluded from the analysis. Test–retest reliability was considered acceptable if ICC > 0.7 (Baumgartner and Jackson, 1999). Criterion validity was examined by Pearson correlations between the measured constructs and PA behavior. Enjoyment scale ratings were recoded (e.g., 1 to 5) because of the negatively worded items.

## Results

Participant age and BMI characteristics by gender in Sample 1 and Sample 2 are presented in Table 1. In the average, these children were just over 10 years of age, and the BMI percentile was above 50%.

Table 2 shows initial CFA model fit criteria for a one-factor model with no correlations across the items in each scale. The CFI for each scale was greater than 0.90. SRMR also suggested acceptable model fit. However, only self-efficacy scale showed acceptable RMSEA.

For the enjoyment scale, error covariances were detected between items 3 and 5, items 2 and 7, and items 1 and 4. The model was thereby modified by setting the mentioned parameters free to vary from their previously fixed values of zero. The final model had acceptable model fit indices [Chi square = 29.74, *df* = 11, RMSEA = 0.08 (90% CI, 0.05–0.11), SRMR = 0.03].

For the social support from family scale, error covariance was detected between items 1 and 2. Items 1 and 2 were similar, but not redundant. The final model with parameters set free to vary had acceptable model fit indices [Chi square = 79.35, *df* = 34, RMSEA = 0.07 (90% CI, 0.05–0.09), SRMR = 0.04].

For the social support from friend scale, error covariances were also detected between items 1 and 2, and items 9 and 10. The model was thereby modified by setting the mentioned parameters free to vary. The final model with such modifications had acceptable model fit indices [Chi square = 65.90, *df* = 33, RMSEA = 0.06 (90% CI, 0.04–0.08), SRMR = 0.04].

The scales' Cronbach's alpha values from Sample 1 are listed in the Table 3, demonstrating acceptable internal consistency reliability. Test–retest reliability for each scale also indicated acceptable values. Bivariate Pearson coefficients between the scales and self-reported PA were all significant and in the expected directions, supporting the criterion validity of the tested scales.

The results from Sample 2 are reported in Table 4. Cronbach's alpha of each scale was greater than 0.7. Only self-efficacy was found to be significantly associated with objectively measured MVPA (*r* = 0.32, *p* < 0.01) and overall PA (*r* = 0.35, *p* < 0.01), and marginally significantly (*r* = 0.21, *p* < 0.06) associated with self-reported MVPA. Enjoyment suggested a trend (*r* = 0.21, *p* = 0.06) to be correlated with objectively measured MVPA, and was significantly associated with overall PA (*r* = 0.23, *p* < 0.05). Only social support (from family and friends respectively) was significantly (*r* = 0.26, *p* < 0.05) associated with self-reported PA in Sample 2.

## Discussion

This study examined the validity and reliability of translated scales previously developed in English to measure PA correlates (self-efficacy, enjoyment, and social support) among healthy weight Hong Kong Chinese children. Although all the items we derived from published instruments, and used among youth, none of the scales have been validated with a young (8–12 years old) Chinese population.

### Factorial validity of the scales

Factorial validity was supported with reasonably good model fit of the one-factor model for each scale, when several items were allowed to covary. The validation studies of the original self-efficacy (Motl et al., 2000) and enjoyment scales (Motl et al., 2001) conducted in US adolescent girls, supported the unidimensionality of these English scales. The validation study of the original social support scale (Sallis et al., 1987) conducted in American college students used exploratory factor analyses instead of CFA, and suggested the 10 items in the present study loaded on one factor. Thus, the present study was consistent with the previous studies and suggested similar structure among Chinese students.

### Criterion validity of the scales

The significant correlations of all scales with self-reported PA (*p* < 0.01) in Sample 1 supported the scales' criterion validity in Hong Kong children.

The self-efficacy scale in the present study showed a moderate correlation with self-reported PA, and the correlation was stronger (0.40 > 0.25) than the previous study (Huang et al., 2011). The same questionnaire (CLASS-C) was used to measure PA in the two studies, and the demographic characteristics of the participants were similar. Thus, items in the present study reflecting more aspects of self-efficacy may explain the stronger correlations. In Sample 2, self-efficacy was the only construct in the study found to be correlated with the objectively measured MVPA. This confirmed that self-efficacy was an important correlate of PA among Chinese children (Huang et al., 2013), and was consistent with the evidence found in western countries (Van der Horst et al., 2007).

PA enjoyment has not previously been studied in Hong Kong children, and showed the smallest correlation with self-reported PA in both samples. The significant correlation with self-reported PA in Sample 1, and marginally significant (*p* = 0.06) correlation with objectively measured MVPA in Sample 2, support examining PA enjoyment as a PA correlate in Hong Kong children with larger samples. In addition, when we used another variable VM cpm, which reflects movements from all vertical, horizontal, and lateral dimensions to represent overall PA, the marginal significant (*p* = 0.06) correlation suggested by the objectively measured MVPA (based on the movement captured from the vertical dimension) became significant (*r* = 0.35, *p* < 0.05).

In contrast to the previous study in which only friend support was correlated with Hong Kong children's PA (Huang et al., 2011), both social support from family and friends correlated with children's self-reported PA, and the influence from family was relatively stronger. In the previous study (Huang et al., 2011), social support from family was assessed by accumulating the agreements of “being physically active with me” from different family members (whole family, father, mother, and siblings). The social support scale in the present study represents more aspects of the construct, which may have resulted in the significant correlation. Peer support may become more important when children approach adolescence (Van der Horst et al., 2007). Therefore, the relative weaker correlation between social support from friends, than from family, and self-reported PA may reflect that most participants were still in the stage where they were influenced more by family than by peers.

### Reliability of the scales

Internal consistency of the tested scales was acceptable. All the items correlated with the corresponding scale with a minimum value of 0.40 (Blunch, 2013). Test–retest reliability of the original English scales has not been reported. In the present study, test–retest reliability of each scale was acceptable.

### Strengths and limitations

This study included a reasonably large sample (Sample 1) to study the psychometrics of the scales. A smaller sample (Sample 2) was included to assess criterion validity using objectively measured PA for the first time among Hong Kong children. There were, however, several limitations. First, both samples (Sample 1 and Sample 2) were convenience samples, which may limit the generalizability of the study. However, the characteristics of participants in the present study were similar with the previous study (Huang et al., 2011), which recruited school children from multiple areas and formed a representative sample. In addition, all three participating schools, were typical Hong Kong Government primary schools, with similar classrooms that served around 30 children, limited outdoor play space, and standard school schedules. Second, we measured children's PA using self-report which suffers numerous limitations. Although objectively measured PA data were collected in Sample 2, the smaller sample size may have limited the statistical power to test the correlations between the variables with PA behavior. In addition, self-reported MVPA was weakly correlated (*r* = 0.27 for boys, *r* = 0.48 for girls) with objectively measured MVPA among Hong Kong children (Huang et al., 2009), which may explain the discrepant correlations between the tested variables and the two measures in the present study. Third, none of the items in the present study were developed specifically for Hong Kong children. A culture-specific measure may be more appropriate for the target population. In addition, studies have suggested that self-efficacy (Ryan and Dzewaltowski, 2002) and social support (Duncan et al., 2005) may be of different types. It may be promising to further develop scales to measure PA self-efficacy and social support of a multidimensional structure among Hong Kong children.

### Implications for future studies

Tests of the sensitivity of these scales to change over time are warranted with longitudinal studies; the magnitude of correlations of these scales with Hong Kong children's PA at different ages would also be important in a more representative sample.

## Conclusion

The present study provided psychometric support for the use of measures of PA self-efficacy, PA enjoyment, and social support for PA among Hong Kong Chinese children with acceptable factorial validity, criterion validity, internal consistency, and test-retest reliability.

## Conflict of interest statement

The authors declare there are no conflicts of interest.

## Figures and Tables

**Table:** 

None	Rarely	A few times	Often	Very often
1	2	3	4	5

**Table 1:** Characteristics of the participants (Hong Kong, 2013–2014).

		Boys	Girls	All
Sample 1	Age	10.2 ± 1.0	10.3 ± 1.0	10.3 ± 1.0
(*n* = 273)	aBMI	18.6 ± 3.5	17.1 ± 2.5	17.8 ± 3.1
	cBMI percentile	63.69 ± 32.67	50.66 ± 30.08	57.23 ± 32.02
Sample 2	Age	10.5 ± 0.7	10.2 ± 0.8	10.4 ± 0.8
(*n* = 84)	bBMI	18.2 ± 3.5	17.8 ± 3.1	18.0 ± 3.4
	cBMI percentile	60.68 ± 32.98	58.89 ± 33.91	59.98 ± 33.15

Values are mean ± SD. BMI = body mass index.

a BMI in Sample 1 was computed by self-reported height and weight data.

b BMI in Sample 2 was computed by the height and weight data measured by researchers.

c BMI percentile was computed by using data from an international sample which included Hong Kong children (Cole and Lobstein, 2012).

**Table 2:** CFA results for each scale (Sample 1, Hong Kong, May–June, 2013).

Scales	χ^2^	DF	*p*	CFI	RMSEA (90%CI)	SRMR
Self-efficacy	45.68	20	0.001	0.94	0.07(0.04–0.10)	0.05
Enjoyment	70.11	14	< 0.001	0.95	0.12(0.09–0.15)	0.04
SSFA	98.48	35	< 0.001	0.93	0.08(0.06–0.10)	0.05
SSFR	144.95	35	< 0.001	0.91	0.11(0.09–0.13)	0.06

SSFA = social support from family. SSFR = social support from friends.

**Table 3:** Internal consistency, test–retest reliability of the scales, and correlations between the measured PA correlates and self-reported PA (Sample 1, Hong Kong, May–June, 2013).

(*n* = 273)	Cronbach's alpha	ICC (95%CI)	*r*
Self-efficacy	0.78	0.88(0.84–0.91)	0.40^a^
Enjoyment	0.90	0.82(0.77–0.86)	0.23^a^
SSFA	0.86	0.86(0.82–0.89)	0.40^a^
SSFR	0.90	0.91(0.88–0.93)	0.35^a^

SSFA = social support from family. SSFR = social support from friends.

a *p* < 0.01.

**Table 4:** Correlations between the measured PA correlates and PA (Sample 2, Hong Kong, 2013–2014).

(*n* = 84)	Cronbach's alpha	*r* (self-reported MVPA)	*r* (objectively measured MVPA)	*r* (VM cpm)
Self-efficacy	0.84	0.21	0.32^b^	0.35^b^
Enjoyment	0.93	0.09	0.21	0.23^a^
SSFA	0.92	0.26^a^	0.07	0.11
SSFR	0.93	0.26^a^	0.02	0.05

SSFA = social support from family. SSFR = social support from friends.VM cpm = vector magnitude counts per minute.

a *p* < 0.05.

b *p* < 0.01.

## Appendix A

**Self-efficacy questionnaire**

1 I can be physically active on most days of the week.
2 I can ask my parent or other adult to do physically active things with me.
3 I can be physically active during my free time on most days even if I could watch TV or play (sedentary) video games instead.
4 I can be physically active on most days even if it is very hot or cold outside.
5 I can ask my best friend to be physically active with me on most days.
6 I can be physically active even at home.
7 I can do active things because I know how to do them.
8 I can be physically active during my free time on most days no matter how busy my day is.

**Enjoyment questionnaire**

When I am active…

1. I feel bored.
2. I dislike it.
3. It's not fun at all.
4. It makes me depressed.
5. It frustrates me.
6. It's not at all interesting.
7. I feel as though I would rather be doing something else.

**Social support questionnaire**

Please rate how often your family members (parents, siblings, relatives, or anyone living in your household) or your friends (classmates, neighbours, or acquaintances) have said or done what is described during the last three months. Please write one number from the following rating scale in each space:

During the past three months, my family or friends:

1. Did PA with me.
2. Offered to do PA with me.
3. Gave me helpful reminders to engage in PA (“Are you going to exercise tonight?”).
4. Gave me encouragement to stick with my PA program.
5. Changed their schedule so we could do PA together.
6. Discussed PA with me.
7. Planned for exercise on recreational outings.
8. Helped plan PA.
9. Asked me for ideas on how they can engage in PA.
10. Talked about how much they like PA.
